# Coronary Sinus Reduction for Refractory Angina Caused by Microvascular Dysfunction—A Systematic Review

**DOI:** 10.3390/jcm15010291

**Published:** 2025-12-30

**Authors:** Mariusz Tomaniak, Adrian Bednarek, Adrian Włodarczak

**Affiliations:** 1 First Department of Cardiology, Medical University of Warsaw, Banacha 1A, 02-097 Warszawa, Poland; 2 Department of Cardiology, The Copper Health Centre (MCZ), 59-301 Lubin, Poland; 3 Faculty of Medicine, Wroclaw University of Science and Technology, 50-981 Wroclaw, Poland

**Keywords:** coronary sinus reducer, coronary microvascular dysfunction, microvascular resistance, coronary physiology, ANOCA

## Abstract

**Background**: Recent observational studies suggest that coronary sinus reducer (CSR) implantation may have a beneficial effect on microcirculatory indices in patients with coronary microvascular dysfunction (CMD). However, to date, there is no comprehensive summary of the evidence regarding the impact of CSR in this population. **Methods**: This systematic review was conducted in accordance with the PRISMA 2020 Statement. The following databases were searched: PubMed, EMBASE, MEDLINE, and ClinicalTrials.gov. Studies assessing microcirculatory indices or primarily involving patients with CMD undergoing CSR implantation were included. **Results**: After the selection process, 17 studies or trials were included in this systematic review. Across observational studies and case series, CSR implantation was associated with significant improvements in coronary microvascular function, including reductions in the index of microvascular resistance and an increase in coronary flow reserve. These physiological changes were accompanied by consistent improvements in angina severity (CCS class), exercise capacity, and quality-of-life measures, particularly in patients with more severe baseline CMD. Evidence was derived mainly from non-randomized studies involving small patient cohorts, with low procedural complication rates. Ongoing randomized trials are expected to clarify the magnitude of benefit and its clinical relevance in this population. **Conclusions**: CSR implantation may offer clinical and physiological benefits in patients with refractory angina due to CMD. However, the lack of randomized evidence and uncertainty regarding long-term effects warrant further adequately powered trials.

## 1. Background

Nearly 50% of patients presenting with anginal symptoms have no obstructive epicardial coronary artery disease [1]. In most cases, these symptoms result from coronary microvascular dysfunction (CMD), which leads to inadequate myocardial oxygen supply and is associated with poorer cardiovascular outcomes and reduced quality of life [1,2]. CMD can develop as a consequence of various underlying pathophysiological mechanisms; evidence suggests that fibrosis, oxidative stress, endothelial dysfunction, decreased vascular density, and excessive contractility of vascular smooth muscle may all contribute to disease development [3], particularly in the presence of comorbid conditions [4]. A wide range of underlying pathomechanisms hampers the development of molecular-targeted pharmacological solutions; thus, direct physiology modification has been introduced as a promising concept.

While optimization of antianginal therapy remains the first-line treatment in patients with angina caused by CMD—a condition previously referred to as a cardiac X syndrome—a substantial proportion of patients suffer from persisting angina symptoms, which severely impairs their quality of life. In this selected patient population, implantation of a stent-like device, namely coronary sinus reducer (CSR), is emerging as a promising option for angina relief.

**Figure 1:**
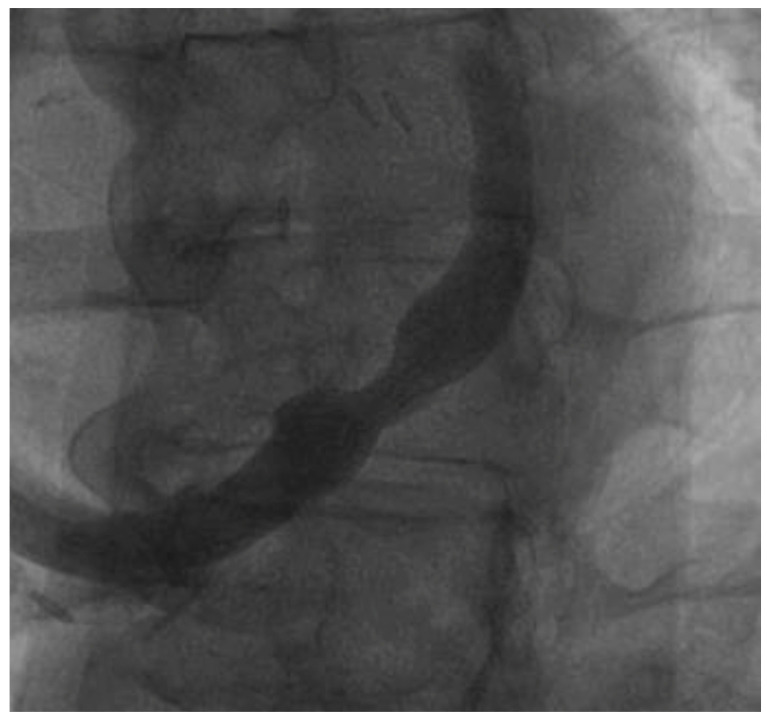
CSR implanted in the coronary sinus in a patient with refractory angina.

CSR narrows the coronary sinus, which causes symptom relief in patients with drug-resistant angina and coronary stenosis (Figure 1). Current data indicate the benefit of CSR implantation in patients with refractory angina, which contributed to the class IIB recommendation for this solution in current guidelines [1]. Several randomized trials, even with a sham control arm, revealed a reduction in angina severity [5,6]. As the implantation of the CSR becomes increasingly available in interventional cardiology centers, it is essential to consider this therapeutic option for patients evaluated for refractory angina, including those seen in an outpatient setting.

This review summarizes current evidence on the impact of coronary sinus modification on biological and physiological coronary function and systematically reviews the data and ongoing trials on CSR implantation effects among patients with refractory angina caused by CMD.

## 2. Current Clinical Solutions for Patients with CMD

Currently, the diagnosis of CMD is primarily based on thermodilution-based measurements, which allow assessment of the index of microcirculatory resistance (IMR) and coronary flow reserve (CFR), as well as on acetylcholine-based coronary vasomotor testing [7]. CMD is diagnosed in the absence of significant epicardial coronary artery stenosis when CFR is below 2.5 or IMR exceeds 25. Current guidelines recommend a treatment algorithm that modifies three main domains—lifestyle modification, risk factors control, and pharmacological therapy [7]. Lifestyle interventions include appropriate nutrition, exercise, weight management, stress control, and smoking cessation. Nevertheless, there are no randomized studies evaluating the effects of any of these interventions in this group of patients. Observational studies showed that CMD presence is significantly associated with hypertension, diabetes mellitus, and dyslipidemia and may develop as their direct consequence [8,9,10,11]. The currently recommended pharmacologic strategy includes beta-blockers, calcium channel blockers, angiotensin-converting enzyme inhibitors, ranolazine, trimetazidine, and ivabradine [7]. It should be highlighted that the data based on randomized trials of these therapies is limited and only applies to the quality of life, not to the objective clinical endpoints. Mentioned pharmaceuticals have a positive pleiotropic effect on the treatment of coronary artery disease rather than a direct effect on microvascular disease. Currently, ongoing trials will deliver the answer on the effectiveness of more precise pharmacological solutions. The PRIZE trial (NCT04097314) is evaluating the endothelin A receptor antagonist (Zibotentan) in CMD, which may mediate the vasoconstrictive effect of endothelin A [12]. Different and not fully understood pathogeneses significantly delay the incorporation of targeted drugs.

Beyond pharmacological treatment, interventional approaches are increasingly being explored to improve angina symptoms and quality of life in patients with CMD. The CSR has been evaluated in several trials, demonstrating significant improvements in angina severity in patients with refractory angina; notably, the COSIRA and ORBITA-COSMIC trials highlighted the therapeutic relevance of coronary sinus narrowing [5,6]. Studies investigating refractory angina have included not only patients with epicardial coronary artery disease but also patients with CMD. Across available CSR studies, patients with CMD have accounted for approximately 5–15% of the studied populations [13,14,15]. Following its success in the treatment of drug-resistant refractory angina, CSR has subsequently been introduced into the studies focusing specifically on patients with CMD. Although no studies have directly compared CSR with microvascular-targeted drugs or other vasomotor-modulating therapies, CSR may serve as an adjunct to standard antianginal therapy in patients who continue to experience refractory symptoms despite optimal pharmacological treatment.

## 3. Coronary Sinus Occlusion—Physiological Perspective

Coronary flow distribution plays a key role in meeting myocardial oxygen demands. Under the conditions of maximal vasodilation, subendocardial perfusion is approximately 50% higher than subepicardial perfusion, reflecting the greater vascular volume in this region [16]. During systole, contraction of intramyocardial and subendocardial arterioles and venules—resulting in a 10–20% reduction in vessel diameter—leads to impaired blood flow in this phase [17]. In contrast, the diameter of subepicardial vessels remains relatively stable, provoking retrograde flow [18,19]. The development of CMD is strongly influenced by microvascular resistance and the distribution of recruited collateral vessels. Pre-arterioles and arterioles are primarily responsible for flow regulation and account for most of the resistance within the coronary microcirculation. In addition to autoregulatory mechanisms, their hyperreactivity may restrict blood flow even under resting conditions, leading to myocardial ischemia. Patients with CMD typically exhibit attenuated vasodilator responses to pharmacological agents, which further impairs flow adaptation and distribution. To date, there are no drugs directly modifying these physiological responses; thus, the device-based strategy has been explored as an alternative therapeutic approach.

Most data on coronary sinus modification and its impact on coronary physiology are derived from studies using temporary balloon inflation. These strategies aim to increase the coronary sinus pressure, which causes the redistribution of the flow to the ischemic region and an increase in the collaterals’ flow through the activation of artero–artero and veno–venous connections [20]. The real mechanisms remain unclear; however, studies have highlighted the potential tremendous role of collateral opening modulation. Experiments performed on pigs, which do not have collaterals, did not reveal the beneficial effect of the retrograde treatment [21,22]. It was also shown that the collateral flow relates to coronary sinus pressure, most likely as a result of the increase in vascular impedance. These modulations are possible due to the multiple connected minor venous systems (cardiac veins, coronary sinus, and Thebesian system) [23]. Studies also suggest that the “sink effect” in which intramyocardial pressure decreases in ischemic regions due to the decreased blood flow, may facilitate the appropriate distribution after the dilation of collaterals [24]. Ido et al. showed that coronary sinus occlusion increases the regional myocardial blood flow in ischemic regions, as well as the index of flow distribution between the subendocardial layer and the subepicardial layer [25]. In addition, sinus occlusion also lowers the index of subendocardial/subepicardial intramyocardial pressure. It should be highlighted that the majority of these studies were performed with the occlusion of the epicardial artery, mimicking acute ischemia rather than the scenario with repetitive ischemia, contributing to the myocardial adaptation as in CMD [25]. Due to the design of basic studies, this solution was clinically tested, mainly in patients with obstructive coronary artery disease. OxAMI-PICSO evaluated the effect of pressure-controlled sinus occlusion (PICSO) in patients with STEMI and a high index of microvascular resistance (IMR) (>40). Patients treated with PICSO had significantly lower IMR values after 24–48 h from stenting and smaller infarct sizes after 6 months compared to the controls [26]. However, it was a single-center, open-label, non-randomized study, which limits the derivation of the conclusions. Subsequent analysis with the control group from OxAMI-PICO showed the differences in CFR, IMR, and resistive resistance ratio (RRR) values in patients with anterior STEMI, depending on the treatment. Nevertheless, patients with inferior STEMI had significant differences only in CFR and RRR values. This supports the theory that coronary sinus occlusion may be beneficial, especially in left anterior descending artery (LAD) occlusion, because the right coronary artery (RCA) territory mainly drains through the Thebesian and cardiac veins system to the right ventricle [27]. Another trial—MACCUS (randomized, sham-controlled)—was conducted in patients with CMD [28]. This study showed that both the resting and hyperemic coronary resistance were significantly lower in patients after the balloon procedure compared to patients with sham procedures [29]. The explorative analysis showed that the decrease in IMR after balloon was largest in patients with the highest sham IMR [29], suggesting the highest benefit in patients with severe CMD.

In turn, CSR implantation allows for constant sinus narrowing, maintaining an elevated pressure. Studies have demonstrated that considerations regarding the beneficial effect of sinus occlusion with CSR based on animal studies are accurate. CSR improves the global myocardial perfusion reserve index, reported especially in ischemic regions [30,31,32]. However, there is no clear answer regarding the transmural flow distribution observed in animals. Interestingly, clinical studies showed that global transmural distribution of the flow (endocardial/epicardial) is not improved by this solution [5,31]; however, studies have reported potential benefits in the ischemic regions. Nevertheless, a recent ORBITA-COSMIC trial showed no difference in stress myocardial blood flow in ischemic segments despite the significant improvement in anginal symptoms [5]. These reports reveal that the real mechanism behind the beneficial effect remains not fully understood. Studies of CSR’s effect on flow and perfusion are mainly based on cardiac magnetic resonance; thus, there is a need for the adoption of other solutions, such as positron emission tomography (PET), to confirm or refute these findings. Limited data on PET-derived analysis are available, suggesting the relation of change of myocardial perfusion reserve (MPR) with the degree of baseline ischemia. Segments with MPR ≥ 1.67 had a significant decrease in MPR after CSR, whereas those with MPR < 1.67 had a significant increase, which supports the theory of the significant redistribution of the flow between myocardial regions [33,34]. More studies in the clinical environment are needed to evaluate the exact physiological mechanism of coronary flow adaptation after CSR implantation. It should be highlighted that most of the studies on flow distribution have been performed on patients after numerous revascularizations, which may not be accurate for patients with CMD.

## 4. Coronary Sinus Occlusion—Molecular Perspective

The coronary flow profile is highly dependent on several autoregulating mechanisms dependent on natural mediators. Microvascular impairment may be caused by an inappropriate balance of mediators or impaired reactions to molecular particles. There have only been a few studies reporting the effects of sinus occlusion on molecular particles expression, and they predominantly represent the PICSO method. It is believed that the pathogenesis of CMD is linked to excessive inflammation. It was shown that patients with CMD have higher levels of CD40-L and C-reactive proteins—both are common inflammatory markers [35,36]. Shurs et al. showed that 17 inflammatory biomarkers are negatively correlated with coronary flow velocity reserve [37]. However, the PICSO was shown to increase the IL-6 concentration, which rather suggests the negative molecular effect of sinus narrowing on microvascular function or represents the stress caused by pulsatile flow due to the repetitive sinus occlusion [38]. It was also reported that permanent occlusion of the venous outflow with subsequent arterialization of the venous coronary vascular compartment stimulates neoangiogenesis [39]. Animal studies have shown that sinus occlusion may upregulate VEGF and heme oxygenase (HO) expression [38]. VEGF is responsible for stimulating neoangiogenesis during homeostasis and disease processes [40], and HO plays a role in cardioprotection [41]. It should be highlighted that upregulation of these factors was revealed in border zones but not in the coronary sinus blood [38,42]. It is postulated that mechanotransduction can be the main factor responsible for the release of neoangiogenesis factors in PICSO; however, the shear stress may also play a significant role. There is a need for the evaluation of biomechanical forces after CSR implantation and correlation with neoangiogenesis factor concentrations to test this hypothesis. In turn, Mohl et al. showed that, in patients with heart failure, PICSO resulted in an increase in miR-19b, miR-101, miR-143, miR-144, and miR-145 and a relative decrease in let-7b, miR-25, miR-191, miR-320b, miR-421, and miR-486-5p [43]. Increased miRs (miR-19b, miR-101, miR-143, miR-144, and miR-145) are known to be involved in muscle development, Wnt pathway, and aortic and coronary endothelial function, which may suggest the beneficial effects of coronary sinus narrowing from a molecular perspective. It should be highlighted that the PICSO method, contrary to CSR, is based on periodic elevation, which may directly influence the expression or molecular washout; thus, similar studies with CSR should be performed to reveal its real molecular effects. To date, there have been no studies evaluating similar parameters after CSR implantation.

## 5. Current Evidence and Ongoing Trials in CMD Population—Systematic Review

**Figure 2:**
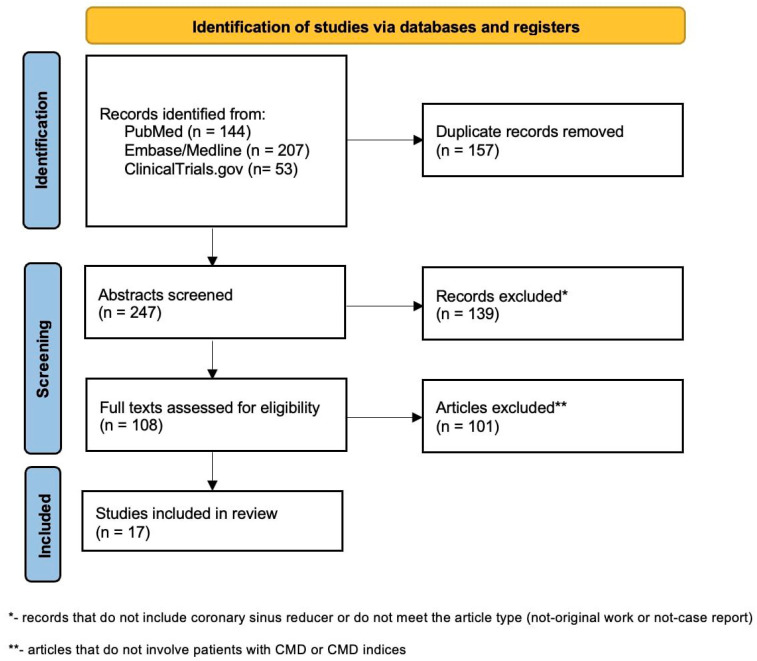
Screening flowchart.

This systematic review was conducted according to the PRISMA 2020 Statement (Table S1). The following databases were searched: PubMed, EMBASE, MedLine, and ClinicalTrials with the terms “coronary sinus reducer” and “coronary sinus narrowing” separately. Articles and trials were extracted independently by two authors; final decisions in arguable cases were reached by consensus between the two screeners. After the removal of duplicates (including removal of conference abstracts that were published later as articles), in the first stage, abstracts and titles screening; non-original works and non-case reports (reviews, meta-analyses, editorials, and responses); and works that do not include CSR were excluded. In the second step, studies involving indices of microcirculation [IMR, CFR, Microvascular Resistance Reserve (MRR), and RRR] or studies focusing mainly on patients with CMD were included (Figure 2). Trials registered in ClinicalTrials were included if they were focused on the evaluation of CSR’s impact in CMD patients or the impact of CSR on the physiological parameters of microcirculation (only trials with results that had not been published yet). A summary of the included articles/trials is presented in Table 1.

### CMD—Coronary Microvascular Disease

The theoretical impact of CSR on microvascular indices has been proven in practice. Giannini et al. reported two cases of the beneficial effect of sinus narrowing on coronary blood flow and microvascular resistance in patients with in-stent restenosis and chronic total occlusion [44]. The first data from an observational study on a larger group of patients were provided by the INROAD study. The trial enrolled 24 symptomatic patients (follow-up available for 21) with maximal tolerated doses of antianginal medications and previous coronary revascularization. The study revealed a significant reduction in IMR assessed after 4 months of follow-up, compared to the baseline (reduction from 33.35 ± 19.88 to 15.42 ± 11.36; *p* < 0.001) [45]. Moreover, there were also significant improvements in CFR (from 2.46 ± 1.52 to 4.20 ± 2.52; *p* = 0.007) and RRR (from 2.81 ± 2.31 to 4.75 ± 2.88; *p* = 0.004) [45]. Apart from physiological indices, significant differences in anginal parameters [Canadian Cardiovascular Society scale (CCS) and Seattle Angina Questionnaire (SAQ) values] were also reported. It should be mentioned that the change in the overall SAQ score, despite its statistical significance, was only around three points in absolute values. Physiological results were most promising in patients with higher IMR values, which may suggest that the scale of benefit depends on the severity of CMD. A study performed by Servoz et al. confirmed the beneficial effect of CSR on microvascular physiological indices and showed that it may be present directly after its implantation. The authors reported a 30% improvement in coronary blood flow (106 ± 41 mL/min before implantation to 139 ± 46 mL/min after implantation; *p* = 0.039) and a 20% reduction in microvascular resistance (796 ± 508 WU to 644 ± 326 WU; *p* = 0.027) [46]. The results of this study should be interpreted with caution because of the small group (10 patients) and the assessment of microvascular function in different vessels (five LAD, three circumflex artery, and two RCA).

Several studies on CSR implantation in patients with CMD have been described so far. Giannini et al. enrolled eight patients with refractory angina and negative fractional flow reserve or stenosis < 50% to implant CSR. It should be highlighted that all patients underwent at least one previous percutaneous coronary intervention [47]. CSR implantation improved anginal symptoms, from median CCS class = 3.0 (3–4) to 1.5 (1–3), *p* = 0.014, with the maintenance of the benefit in three of the five patients assessed and discontinuation of at least one antianginal medication in 37.5% of patients [47]. Significant improvement was also seen in the 6-min walking test, quality of life, and symptoms reported with SAQ [47]. To date, only results of small observational studies and case reports with confirmed microvascular parameters have been published. Among patients from the COMPLEX registry, five patients were confirmed to have CMD, with a mean IMR = 32.8 ± 15.7 and CFR = 1.7 ± 0.7 [48]. After a median follow-up of 647.5 days after CSR implantation, all patients improved by ≥1 CCS point, with only one procedure-related complication (sternocleidomastoid muscle hematoma) [48]. A similar trend has also been observed in other studies; Konigstein et al. described the results of CSR implantation in 23 patients with angina with nonobstructive coronary artery disease (ANOCA) [49]. After four months, there were improvements in IMR (31 ± 10 to 22 ± 16; *p* = 0.02) and CFR (1.7 ± 0.5 to 2.9 ± 1; *p* = 0.001), as well as CCS class, 6-min walk test, and all domains of the SAQ [49]. Also, the results of the novel self-expanding A-FLUX device (VahatiCor, Santa Clara, CA, USA) suggest the potential utility of this solution in patients with CMD [50]. A dedicated study for patients with ANOCA was also conducted at the Mayo Clinic. Neovasc CSR (Shockwave Medical, Santa Clara, CA, USA) was implanted in 30 patients, which resulted in a significant improvement in CFR measured at 120 days (2.1, 1.95–2.30 vs. 2.7, 2.45–2.95; *p* = 0.0019) [51]. There was also a reduction in hyperemic microvascular resistance and improvement in the CCS class and the SAQ results [51]. Two safety endpoints were reported (wire-related perforations), and both patients required pericardiocentesis. Although detailed links between mechanistic improvements and hard clinical outcomes cannot be established yet due to limited data, improvements in CFR may plausibly contribute to a reduction in major adverse cardiac events; nevertheless, an adequately powered clinical trial is needed to evaluate this conception [2].

In turn, Hoole et al. reported a case of patients with hypertrophic cardiomyopathy and coronary microvascular disease with refractory angina despite three antianginal medications. The implantation of CSR led to the improvement of global stress myocardial blood flow and anginal symptoms [52]. Other described cases have revealed similar effects [53,54]. Previous CSR trials in refractory angina excluded patients requiring resynchronization therapy; nevertheless, there have been individual attempts on the feasibility of this intervention in patients with CSR, including patients with CMD [55].

It should be highlighted that the current evidence is not sufficient to recommend CSR implantation in patients with CMD; however, the results of ongoing randomized trials will provide valuable data on the utility of coronary sinus narrowing in those patients. There are two randomized controlled trials ongoing, namely REMEDY-PILOT and COSIMA [56,57]. The COSIMA trial plans to enroll 144 patients with CCS 3–4 and evidence of CMD defined by IMR > 25 or CFR < 2.0 [57]. Patients are randomized by CSR implantation (Neovasc) or only optimal medical therapy, and the primary outcome is improvement ≥2 CCS angina classes at six months [57]. REMEDY-PILOT will recruit 54 patients with CCS Class 2–4 for ≥3 months despite maximal therapy without epicardial stenosis (<30%) and with subendocardial hypoperfusion detected on CMR [ischemia with no obstructive coronary arteries (INOCA) patients] [56]. Patients are randomized by the implantation of the Neovasc reducer or a sham procedure. The primary endpoint includes a change in global myocardial perfusion reserve, but other outcomes will also assess physical endurance, anginal symptoms, and perfusion distribution. In addition, the recently launched REDUCE-ANOCA study (NCT07010029) will recruit 50 patients with CMD-related ANOCA, testing the impact of the Neovasc reducer on coronary function and echocardiographic parameters, on top of angina assessment. Of note, there is also a randomized COSIRA-II study ongoing; however, patients with CMD are included only in the third arm, which is based on registry without randomization [58]. A-FLUX (VahatiCor, Santa Clara, CA, USA) self-expandable coronary sinus reducer is currently being tested in the ongoing SERRA-I early feasibility study (NCT06991322). There are also other ongoing trials focused on CSR’s impact on myocardial perfusion and microvascular physiological indices [59,60].

## 6. Limitations and Challenges

Still, several limitations for wider CSR use in patients with CMD need to be addressed. The most important is the limited amount of data, especially the lack of data from sham-controlled, randomized clinical trials in patients with CMD. COSIMA and REMEDY-PILOT will provide results on CSR clinical utility; however, the total number of randomized patients is less than 200. The studies included in this review are predominantly small, single-center studies or case series; therefore, all reported findings should be interpreted with caution, given the limited number of participants. Given the small sample sizes and the predominance of observational studies included in this review, the possibility of publication bias cannot be excluded and should be considered when interpreting the results. Moreover, current trials typically exclude patients with right atrial pressure greater than 15 mmHg; thus, findings and recommendations cannot be extrapolated to this group. There is also a need for the identification of specific CMD profiles that may benefit from CSR. According to the data, the most beneficial effect may be present in patients with high IMR values; however, the optimal cut-off for IMR, CFR, and MRR should be defined to enable a precise benefit-to-risk estimation. The problem may also be caused by the small variety of devices.

Despite several hypotheses, the exact mechanism of CSR action is still unclear. Data on the effects of CSR on the molecular environment are limited. In addition, findings from physiological studies, particularly those related to flow distribution, remain inconsistent. Before this solution can be introduced into routine clinical practice, the mechanisms underlying its beneficial effects must be clearly defined. Furthermore, concerns have been raised regarding the long-term effectiveness of CSR implantation. One possible explanation for reduced efficacy is increased activity in alternative venous drainage pathways following implantation. Some evidence suggests that this process may contribute to the lack of response observed in certain patients [61]. However, further studies are required to identify the predictors of lower response to this device therapy.

## 7. Conclusions

CSR implantation is a promising strategy for patients with refractory angina caused by CMD. While results from ongoing randomized clinical trials are needed to establish its value in daily clinical practice, it is important to note that no randomized controlled trial has yet been completed specifically in CMD and that the underlying mechanisms and long-term durability of the benefits remain under investigation. Nevertheless, several reports suggest beneficial effects on the microvascular physiological parameters and flow distribution, translating into angina relief and improved quality of life.

## Figures and Tables

**Table 1:** Summary of the included studies.

DOI/NCT Identificator	First Author or PI	Study Type	Population	Design	Country	Device	Outcomes
10.1016/j.jcin.2017.06.062	F. Giannini	Observational	8 patients with Evidence of complete revascularization and non-obstructed epicardial coronary arteries	Observation from baseline to follow-up	Italy	Neovasc	Improvement of CCS class from 3.0 (3–4) to 1.5 (1–3), *p* = 0.014. Improvement in SAQ values and 6-min-walk test—266 m (238.5–372.8 m) to 360 m (341–420 m), *p* = 0.018 and Borg scale from 4.0 (3–5) to 0.0 (0–2.5), *p* = 0.042 In subgroup of three patients—significant improvement of MPRI
NCT04606459	T. Gori	RCT	144 patients with Refractory angina CCS class III-IV despite guideline-directed medical therapy Evidence of reversible ischemia on non-invasive testing Evidence of microvascular disease as diagnosed invasively (IMR > 25 or CFR < 2.0 with FFR > 0.8)	CSR implantation or optimal medical therapy—assessment at 6 months, 1 year and 5 years	Germany	Neovasc	Primary endpoint—change in CCS Other endpoints include: SAQ values, procedural success, EQ-5D-5L, use of nitrates, unplanned revascularization, death, MI, ED visit, procedural complications
NCT05102019	T. D. Henry; G. W. Stone	CMD only in registry arm	380 patients in total three arms (RCT + registry). In registry arms patients including: Patients with non-obstructive CAD (coronary narrowing of <50%, and/or FFR ≥ 0.81)	Registry arm with CSR implantation—maximum follow-up at 5 years	United States and Canada	Neovasc	Primary endpoints: Change in exercise duration in a modified Bruce treadmill Safety events: death, MI, pericardial effusion, device embolization, BARC 3 or 5 Secondary endpoints: CCS and SAQ
NCT05492110	R. E. Silva	RCT—sham-controlled, double-blinded	54 patients with INOCA: Ongoing symptomatic angina, CCS Class II-IV, for ≥3 months despite background treatment with at least two anti-anginal drug at the maximal tolerated dose. Unobstructed coronary arteries with ≤30% epicardial stenoses demonstrated on coronary angiography. Circumferential subendocardial stress-induced hypoperfusion on CMR (global MPR < 2.5)	CSR implantation or sham-controlled—outcomes at 6 months	England	Neovasc	Primary endpoints: Change in myocardial perfusion Secondary endpoints: CCS, SAQ, SF-36, HADS, 6MWT, Borg scale and safety events Other endpoints: Perfusion distribution
10.1093/ehjcr/ytac440	K. Cheng	Case Report	38-year-old women with chest pain despite multi anti-anginal drugs, and ischemia probably due to the CMD in hypertrophic cardiomyopathy	-	England	-	At 6 months follow-up CCS1 and change in SAQ (+14 points), as well as the reduction of ischemic burden from 16% to <5%
10.1093/ehjcr/ytac159	F. Giannini	Case Report	Two patients: 58-year-old male after CABG and numerous PCI and 78-year-old man with CTO. Both patients were not suitable for revascularization and on optimal therapy	-	Italy	Neovasc	Increase in CBF—from 100 to 148 mL/min for the first patient and from 107 to 133 mL/min for the second patient and decrease of microvascular resistance from 516 to 362 woods units for the first patient and from 543 to 478 woods units for the second patient.
NCT06033495	Ø. Lie	Observational	15 patients with: Coronary artery disease and refractory angina Clinical indication for coronary sinus reduction stent implantation	CSR implantation and 15O-H2O PET/CT assessment at six months	Norway		Primary outcome: Change in myocardial flow reserve on 15O-H2O PET/CT Secondary outcome: SAQ, QoL, Exercise capacity and CFR
10.1093/eurheartj/ehad655.1296	E. Gnan	Observational	8 patients with: Absence of obstructive coronary artery disease (<50% narrowing in all coronary arteries, or a negative intracoronary fractional flow reserve test) and no prior history of revascularization 5 patients (62.5%) had confirmed CMD	CSR implantation and observation for a median time of 647.5 (132–732) days	Switzerland		Improvement of CCS (2.9 ± 0.6 to 1.5 ± 0.8) and number of antianginal drugs (2.6 ± 1.6 to 1.9 ± 1.1)
10.1093/ehjcr/ytad455	C. Grebmer	Case report	Patient with microvessel disease with therapy refractory angina and non-relevant stenosis of RCA	-	Switzerland		Feasibility of cardiac resynchronization therapy after CSR implantation
NCT06266065	J. Bulum	Observational	25 patients with: Coronary artery disease and refractory angina pectoris who are ineligible for coronary revascularization	CSR implantation and observation for 2.5 years	Croatia		Primary endpoint: CFR and IMR at 2.5 years Secondary endpoints: Evaluation of the ischemic zone on CMR, SAQ and 6-min-walk test
10.1016/j.cjco.2024.07.011	S. P. Hoole	Case report	69-year-old woman with hypertrophic cardiomyopathy diagnosed with NSTEMI without obstruction of coronary artery	-	England	Shockwave	Improvement of CCS—3 to 2, global sMBF (1.14 mL/min/g to 1.5 mL/min/g) and MPR (1.9 to 2.0)
10.1016/j.jacc.2024.09.798	M. Konigstein	Observational	23 patients with: Refractory angina and microvascular dysfunction	CSR implantation and observation for 4 months	Israel	Neovasc	Improvement in CCS class (from 3 to 2), CFR (1.7 ± 0.5 to 2.9 ± 1, *p* < 0.001), IMR (31 ± 10 to 22 ± 16, *p* = 0.02), 6MWT (303 to 345, *p* = 0.04) and all SAQ domains (*p* < 0.01)
10.1016/j.jacc.2024.09.816	J. M. Paradis	Observational	11 patients including: 5 patients with CMD 6 patients with obstructive CAD	CSR implantation and observation for 6 months		A-Flux	Improvement in CCS class and all SAQ domains at 30 days, 3 months and 6 months (*p* < 0.01)
10.33963/v.kp.98104	P. Rola	Case report	65-year-old male patient with CCS3 despite 6 months of optimal anti-anginal therapy with CMD	-	Poland		Improvement in CFR (2.2 to 4.1), IMR (46 to 11), CCS class (3 to 1), 6MWT (90 to 300 m), SAQ, EQ-5D, SF-36
10.1002/ccd.31070	C. Servoz	Observational	10 patients with: Refractory angina	CSR implantation and direct measurements after	France	Shockwave	Improvement in maximal absolute coronary flow (106 ± 41 to 139 ± 46, *p* = 0.039), minimal microvascular resistance (796 ± 508 to 644 ± 326, *p* = 0.027) and CCS class (3.4 ± 0.5 to 1.7 ± 1.0, *p* = 0.004—after one month)
10.1161/circinterventions.123.013481	M. Tebaldi	Observational	24 patients with: Refractory angina At least one opened coronary artery (excluding RCA)	CSR implantation and observation for 12 months	Italy	Neovasc	Improvement in primary endpoint—IMR (33.35 ± 19.88 to 15.42 ± 11.36, *p* < 0.001), CFR (2.46 ± 1.52 to 4.20 ± 2.52, *p* = 0.007), RRR (2.81 ± 2.31 to 4.75 ± 2.88, *p* = 0.004), CCS class, and SAQ angina frequence, angina stability, QoL and summary score
10.1016/j.jcin.2024.09.018	D. Tyron	Observational	30 patients with: ANOCA CMD CCS class 3–4 despite maximally tolerated therapy	CSR implantation and observation for 120 days	United States	Neovasc	Improvement in CFR in response to adenosine [2.1 (1.95–2.30) to 2.7 (2.45–2.95), *p* = 0.0019], percent change in coronary artery blood flow in response to acetylcholine [11.0 (20.15 to 5.85) to 11.5 (4.82 to 39.29), *p* = 0.0420], hyperemic microvascular resistance [1.45 (1.05–1.98) to 1.86 (1.44–2.35), *p* = 0.0420], CCS class [4.0 (3.25–4.0) to 2.0 (2.0–3.0), *p* < 0.001] and SAQ results (all *p* < 0.01)

6MWT—6-Minute Walk Test, ANOCA—Angina with No Obstructive Coronary Arteries, CABG—Coronary Artery Bypass Grafting, CAD—Coronary Artery Disease, CBF—Coronary Blood Flow, CCS—Canadian Cardiovascular Society (classification of angina), CFR—Coronary Flow Reserve, CMD—Coronary Microvascular Dysfunction, CSR—Coronary Sinus Reducer, CTO—Chronic Total Occlusion, FFR—Fractional Flow Reserve, IMR—Index of Microcirculatory Resistance, INOCA—Ischemia with No Obstructive Coronary Arteries, MPR—Myocardial Perfusion Reserve, PET—Positron Emission Tomography, QoL—Quality of Life, RCT—Randomized Controlled Trial, SAQ—Seattle Angina Questionnaire, and sMBF—Stress Myocardial Blood Flow.

## Data Availability

No new data were generated.

## Supplementary Materials

The following supporting information can be downloaded at https://www.mdpi.com/article/10.3390/jcm15010291/s1: Table S1 PRISMA checklist.
